# The interaction between the gene SOD1 rs2070424 and the plasma zinc/copper ratio predicts renal function impairment

**DOI:** 10.3389/fnut.2025.1716103

**Published:** 2025-11-27

**Authors:** Jingsi Chen, Jiaju Ge, Jiayuan Song, Tianci Wang, Yuqin Ma, Xiaoyan Qian, Chang Zhou, Qiuguo Wang, Senlin Zhu, Xiyu Wu, Hongzhen Du, Liqiang Qin, Zengning Li

**Affiliations:** 1 Department of Nutrition and Food Hygiene, Suzhou Medical College of Soochow University, Suzhou, China; 2 Suzhou Industrial Park Centers for Disease Control and Prevention, Suzhou, China; 3 Department of Nutrition, The First Hospital of Hebei Medical University, Shijiazhuang, China; 4 Hebei Key Laboratory of Nutrition and Health, Shijiazhuang, Hebei, China; 5 Hospital of Stomatology, Hebei Medical University, Shijiazhuang, China

**Keywords:** copper, renal function, superoxide dismutase 1, zinc, zinc/copper ratio

## Abstract

**Background:**

Essential trace elements zinc and copper have been suggested to play a role in renal function. However, the balance between these elements and their interaction with genetic variation in *Superoxide Dismutase 1* (*SOD1*) remains unclear.

**Methods:**

We conducted a prospective study involving 1,274 middle-aged and elderly participants from the “135” cohort in 2015. Cox regression analyses were used to assess associations between plasma trace elements, *SOD1* polymorphism (rs2070424), and their interactions with impaired renal function. To assess predictive performance, we compared discrimination and reclassification using ROC analysis, as well as the net reclassification index (NRI) and integrated discrimination improvement (IDI).

**Results:**

After adjusting for multiple variables, the hazard ratios (HRs) (95% CI) comparing the highest to the lowest tertiles were 1.303 (1.037, 1.637) for zinc, 1.615 (1.275, 2.046) for the zinc/copper ratio, and 0.709 (0.558, 0.900) for copper. Compared with the GG genotype, the AG and AA genotypes were positively associated impaired renal function (HR: 1.282, 95% CI: 1.003, 1.639). Positive associations between impaired renal function and plasma zinc (*P* for interaction: 0.006), as well as the zinc/copper ratio (*P* for interaction: <0.001), were modified by rs2070424 genotypes. The adjusted HRs comparing tertile 3 with tertile 1 for impaired renal function were: AA/AG carriers—zinc 1.359 (1.055–1.749); zinc/copper ratio 1.642 (1.267–2.128); GG carriers—zinc 1.065 (0.623–1.819); zinc/copper ratio 1.584 (0.889–2.822). In prediction analyses, the zinc/copper ratio showed higher discrimination (AUC) than single-metal models and improved reclassification (NRI, IDI), providing partial evidence of incremental value.

**Conclusion:**

Decline in renal function was positively correlated with plasma zinc and the zinc/copper ratio and negatively correlated with plasma copper. The relationship between impaired renal function and both zinc and the zinc/copper ratio was modified by rs2070424 genotypes. Taken together, considering zinc/copper ratio provides a more informative summary than single-metal measures and shows partial incremental discrimination.

## 1 Introduction

Chronic kidney disease (CKD) has emerged as a major global health concern. The Global Burden of Disease (GBD) project estimated that in 2017, approximately 697.5 million people were affected by CKD across all stages, corresponding to a global prevalence of 9.1% (8.5%–9.8%) (1). A 2021 joint report by the American Society of Nephrology, the European Renal Association, and the International Society of Nephrology further highlighted that more than 850 million individuals worldwide live with kidney disease—about twice the number of those with diabetes and 20 times that of cancer or HIV/AIDS. These figures underscore the escalating burden of kidney disease and its growing importance as a public health priority.

Despite its widespread prevalence, the etiology of CKD is multifactorial and remains only partially understood. Beyond traditional risk factors such as hypertension and diabetes, accumulating evidence suggests that both environmental exposures and genetic susceptibility substantially contribute to renal pathophysiology. Among environmental determinants, nutritional and trace element status have attracted increasing attention (2), while genetic variants involved in oxidative stress defense pathways are increasingly recognized as potential modifiers of renal injury risk (3).

Trace elements such as zinc and copper are essential for numerous enzymatic and antioxidant processes (4). Disturbances in their homeostasis may result from impaired renal excretion or metabolic alterations (5). Zn acts as a cofactor for over 300 enzymes and exerts anti-inflammatory, antioxidant, and immunomodulatory effects (6), whereas Cu plays vital roles in red blood cell formation, mitochondrial energy production, and neuropeptide synthesis (7, 8). Although epidemiological studies have investigated the associations between circulating zinc or copper and renal function, findings remain inconsistent (9–11). Because these two metals compete for absorption and binding sites, an imbalance in their ratio can lead to physiological dysfunction. The zinc/copper ratio has therefore been proposed as a more reliable biomarker of trace element homeostasis and metabolic health than either element alone (12–14). Nevertheless, the relevance of zinc-copper balance to renal function impairment has not been adequately explored.

Both zinc and copper are cofactors of superoxide dismutase 1 (SOD1), a key antioxidant enzyme that catalyzes the dismutation of superoxide radicals into hydrogen peroxide and oxygen (15). SOD1 is central to maintaining redox homeostasis, and its dysregulation contributes to oxidative stress–related disorders, including CKD (16). Experimental studies have shown that Cu supplementation can enhance SOD1 activity and mitigate renal oxidative injury in animal models (17). In human populations, common SOD1 polymorphisms have been associated with kidney disease and diabetic nephropathy (18). However, potential interactions between trace element balance (zinc/copper ratio) and SOD1 genetic variation in the development of renal dysfunction have not yet been systematically evaluated.

To address this gap, we conducted a 4-year prospective cohort study among middle-aged and elderly residents in Suzhou Industrial Park, China. This study aimed to (i) investigate the associations of plasma zinc, copper, and zinc/copper ratio with renal function, and (ii) examine the gene–environment interaction between SOD1 rs2070424 polymorphism and trace element status in predicting renal function impairment.

## 2 Method

### 2.1 Study population

We analyzed data from the “135” cohort, an ongoing prospective study conducted in the Industrial Park of Suzhou, China. Details of the study design and participant characteristics have been previously published (19, 20). Briefly, the “135” survey has been conducted biennially since 2013. The blood samples collected in 2013 were inadequate for trace metal analysis, so data from 2015 was used as the baseline for this study. A total of 4,350 participants aged ≥45 years completed the baseline survey, with 2,487 individuals returning for the follow-up survey in 2019. After excluding 49 participants without demographic or disease information, 363 with malignant tumors, kidney disease, or severe mental illness at baseline, 707 with insufficient blood samples for metal measurement, and 155 lacking genotyping data, 1,274 subjects remained for the final analysis (Supplementary Figure 1). All participants gave written informed consent prior to participation. The study followed the guidelines of the Declaration of Helsinki and received approval from the Ethics Committee of Soochow University (ECSU-2010-002).

### 2.2 Measurement of plasma zinc and copper levels

Plasma concentrations of zinc and copper were determined using an inductively coupled plasma mass spectrometer (ICP-MS), as described by Zhang et al. (21). In brief, 0.3 mL of plasma was placed into digestion tubes and combined with 0.5 mL of 65% nitric acid (HNO₃), 1 mL of 30% hydrogen peroxide (H₂O₂), and 5 mL of ultrapure water. The resulting mixture was digested for 65 min. After digestion, the residue was dissolved in 10 mL of ultrapure water, filtered through a 0.22 μm filter, and analyzed using ICP-MS (NexION™350X, PerkinElmer Inc., USA).

During measurement, Samples were processed in random order to ensure the effectiveness of the blind method. Spiked pooled plasma samples were used to assess method precision and accuracy. Recoveries were 89% for Zn and 99.7% for Cu; RSDs were 1.2 and 2.0%, respectively (Supplementary Table 1). Metal concentrations below the limits of detection (LOD) were reported as LOD/2.

### 2.3 Assessment of impaired renal function

Serum creatinine concentrations were determined automatically using an Olympus AU640 autoanalyzer (Olympus, Kobe, Japan). The analysis was carried out by physicians adhering to established laboratory protocols and quality control standards. Impaired renal function was defined as meeting either of the following: incident CKD (follow-up eGFR <60 mL/min/1.73 m^2^ among participants with baseline eGFR ≥60 mL/min/1.73 m^2^) or rapid decline in renal function (a two-point annualized loss in eGFR of at least 5 mL/min/1.73 m^2^ per year between the two study measurements).

### 2.4 Genotyping

Based on previously identified *SOD1* polymorphism loci (3) associated with kidney disease, single nucleotide polymorphisms (SNPs) with minor allele frequencies (MAF) above 0.05 were selected using HapMap HCB and CHB datasets from dbSNP.^1^ To explore the association between *SOD1* and renal function impairment, two key *SOD1* SNPs (rs2070424 and rs1041704) were chosen.

SNP genotyping was performed using the ligase detection reaction and run on Applied Biosystems (ABI3730XL Hitachi Genetic Analyzer, USA). Genomic DNA was extracted from peripheral blood samples of 1,274 individuals using a Qiagen kit (Germany). Genotyping of both SNPs was successfully performed, with a call rate greater than 95%. However, only the genotype distribution of rs2070424 satisfied Hardy–Weinberg equilibrium (*p* > 0.05) in the baseline cohort (data not shown). Consequently, rs1041704 was excluded from the analysis.

### 2.5 Statistical analysis

All data analyses were performed using SPSS 20.0 (SPSS Inc., Chicago, IL) and R (v4.3.3). Normally distributed continuous variables are presented as mean (SD), while non-normally distributed ones are shown as median (IQR). Categorical variables are expressed as counts and percentages. The appropriate statistical tests were used: the Student’s *t*-test or Mann–Whitney *U*-test for continuous data based on their distribution, and the chi-square test for categorical data. Plasma levels of zinc, copper, and the zinc/copper ratio were log-transformed due to skewness. A *p*-value of <0.05 was considered statistically significant.

Plasma copper, zinc, and zinc/copper ratio, were categorized into tertiles (T1, T2, T3) and analyzed in Cox regression models, using the lowest tertile (T1) as the reference. Hazard ratios (HRs) and 95% confidence intervals (CI) were adjusted for potential confounders, including age, gender, BMI, smoking status, and alcohol consumption. The proportional hazards assumption was verified using Schoenfeld residuals. To further explore the associations, restricted cubic splines with four knots at the 20th, 40th, 60th, and 80th percentiles were applied in R (Harrell, F. E. rms: Regression Modeling Strategies). The reference value was set at the 10th percentile, curves were truncated to the 5th–95th percentiles to limit extrapolation.

Hardy–Weinberg equilibrium for gene genotypes was evaluated using a likelihood ratio test. Cox regression analysis was used to determine the hazard ratio (HR) and 95% confidence interval (CI) for kidney impairment linked to rs2070424 polymorphisms (GG vs. AG/AA genotypes). Additionally, we assessed the joint effect of plasma metal concentrations (Tertiles) and rs2070424 polymorphisms on kidney impairment. Interaction effect was assessed on the multiplicative scale by adding a cross-product term between the continuous exposure (natural-log transformed and standardized per SD) and genotype (dominant model: AA/AG vs. GG) in the Cox regression.

We assessed the performance of Zinc, Copper and Zinc/Copper ratio (log10-transformed and modeled as continuous predictors) by evaluating their discriminative capabilities using the receiver operating characteristic (ROC) curve and calculating the area under the ROC curve (AUC). Subsequently, we employed the net reclassification index (NRI) and integrated discrimination improvement (IDI) index to further assess the incremental predictive value of Zinc/Copper ratio compared to Zinc or Copper individually. Finally, the clinical benefits were compared using decision curve analysis (DCA). Which plots net benefits against different threshold probabilities (22). In the decision curve, a reference line called the treat-for-all scheme represents the maximum clinical costs, while a reference line named the treat-for-none scheme indicates no clinical benefit. A decision curve positioned further away from these reference lines suggests a greater clinical value of the prediction variable.

## 3 Result

### 3.1 Baseline characteristics

Table 1 presented the baseline characteristics of the 1,274 participants included in this study. The average age was 61.7 years, and 56.2% of the participants were male. Compared to individuals with normal renal function, those with eGFR < 60 mL/min/1.73 m^2^ and rapid kidney function decline had higher baseline levels of serum creatinine, total cholesterol, and low-density lipoproteins, along with lower levels of high-density lipoproteins. Participants with impaired renal function exhibited significantly higher zinc concentrations and zinc/copper ratios, as well as lower copper levels, compared to those with normal renal function.

**Table 1:** Comparison of demographic characteristics, baseline physical and biochemical parameters, and plasma metal concentrations between normal and impaired renal function participants.

Parameters	Baseline	Impaired renal function		
		Yes	No	*P*
Participants, *n* (%)	1,274	438 (34.4)	836 (65.6)	
Age (years)	61.7 ± 8.9	61.7 ± 8.8	61.6 ± 8.9	0.185
Gender, *n* (%)				0.185
Male	716 (56.2)	235 (18.4)	481 (37.8)	
Female	558 (43.8)	202 (15.9)	356 (27.9)	
Smoking status, *n* (%)				0.341
Never smoking	748 (58.7)	266 (20.9)	482 (37.8)	
Current smoking	455 (35.6)	145 (11.4)	310 (24.3)	
Former smoking	71 (5.6)	27 (2.1)	44 (3.5)	
Alcohol consumption, *n* (%)				0.297
Frequent drinking	202 (15.8)	74 (5.8)	128 (10.0)	
Occasional drinking	164 (12.9)	48 (3.8)	116 (9.1)	
Never drinking	908 (71.3)	316 (24.8)	592 (46.5)	
BMI (kg/m^3^)	23.8 ± 3.1	23.9 ± 3.9	23.8 ± 3.1	0.497
Systolic blood pressure (mm Hg)	127.3 ± 16.1	125.6 ± 15.5	127.6 ± 14.9	0.039
Diastolic blood pressure (mm Hg)	79.0 ± 11.1	78.7 ± 10.1	78.6 ± 10.4	0.639
Serum creatinine (μmol/L)	71.9 ± 27.3	74.7 ± 24.6	69.7 ± 14.2	<0.001
FBG (mmol/L)	5.8 ± 1.4	5.9 ± 1.6	5.8 ± 1.3	0.661
Total cholesterol (mmol/L)	4.8 ± 0.9	4.9 ± 0.9	4.7 ± 0.8	<0.001
Triglycerides (mmol/L)	1.4 ± 1.1	1.4 ± 0.9	1.4 ± 1.1	0.918
HDL-C (mmol/L)	1.1 ± 0.3	1.1 ± 0.2	1.2 ± 0.2	<0.001
LDL-C (mmol/L)	3.1 ± 0.8	3.2 ± 0.9	3.0 ± 0.7	<0.001
Zn (μg/L)	2402.9 (1904.3, 3209.9)	2514.3 (1940.9, 3358.9)	2331.3 (1866.1, 3119.9)	0.004
Cu (μg/L)	1130.9 (968.2, 1328.8)	1107.1 (935.4, 1275.9)	1144.5 (987.2, 1348.3)	0.087
Zn/Cu ratio	2.1 (1.6, 2.8)	2.3 (1.7, 3.2)	2.0 (1.6, 2.7)	<0.001

### 3.2 The association between metal concentrations and impaired renal function

During the 4-year follow-up, 438 participants developed impaired renal function, including 178 with eGFR < 60 mL/min/1.73 m^2^ and 385 with rapid kidney function decline. After multivariate adjustment (model 2), plasma zinc and the zinc/copper ratio were positively associated with the risk of impaired renal function, while plasma copper was negatively associated. The hazard ratios (HRs) and 95% confidence intervals (CIs) were calculated to compare the highest and lowest tertiles with 1.303 (1.037, 1.637) for zinc, 1.615 (1.275, 2.046) for the zinc/copper ratio, and 0.709 (0.558, 0.900) for copper (Table 2). In the spline regression model, the risk of impaired renal function increased significantly with higher zinc/copper ratios, as well as lower copper concentrations (Supplementary Figure 2).

**Table 2:** The association between tertiles of metals concentration and impaired renal function.

Variables	Tertiles of plasma metals’ concentration			*p* value for trend	*P*
	T1	T2	T3		
*Zinc*					
Case/control	133/291	133/292	172/253		
Crude	1	0.998 (0.785, 1.269)	1.290 (1.029, 1.618)	0.020	0.033
Model 1	1	1.008 (0.792, 1.283)	1.309 (1.042, 1.644)	0.015	0.026
Model 2	1	1.008 (0.792, 1.284)	1.303 (1.037, 1.637)	0.017	0.030
*Copper*					
Case/control	165/259	150/274	123/303		
Crude	1	0.909 (0.729, 1.134)	0.742 (0.587, 0.937)	0.012	0.041
Model 1	1	0.887 (0.709, 1.109)	0.716 (0.565, 0.909)	0.006	0.022
Model 2	1	0.884 (0.706, 1.106)	0.709 (0.558, 0.900)	0.005	0.017
*Zinc/Copper*					
Case/Control	119/305	136/289	183/242		
Crude	1	1.140 (0.892, 1.458)	1.534 (1.218, 1.933)	<0.001	<0.001
Model 1	1	1.168 (0.912, 1.496)	1.608 (1.270, 2.037)	<0.001	<0.001
Model 2	1	1.175 (0.917, 1.506)	1.615 (1.275, 2.046)	<0.001	<0.001

Model 1 was adjusted for sex, age (years), and body mass index (kg/m^2^). Model 2 was adjusted for Model 1 entering smoking (current, former and never) and drinking (frequent, occasional, and never).

### 3.3 The association between rs2070424 polymorphism and impaired renal function

The frequencies of AA and GA genotypes in the normal renal function and impaired renal function groups were 636 (63.9%) and 360 (36.1%), respectively. After multivariate adjustment, the AG and AA genotypes were positively associated with the risk of impaired renal function, with a hazard ratio of 1.282 (1.003, 1.639) compared to the GG genotype (Table 3).

**Table 3:** The association between rs2070424 polymorphism and impaired renal function.

rs2070424 genotype	Controls (%)	Cases (%)	Crude HR(95%CI)	*P* value	Adjusted HR(95%CI)	*P*
GG	200 (71.9)	78 (28.1)	1		1	
AA+GA	636 (63.9)	360 (36.1)	1.274 (0.997, 1.628)	0.053	1.282 (1.003,1.639)	0.047

### 3.4 Interactions between metals concentrations and rs2070424 polymorphism on impaired renal function

On the multiplicative scale, rs2070424 modified the associations of both zinc and Zn/Cu with impaired renal function (P-interaction <0.05; Table 4). In AA/AG carriers, zinc and zinc/copper ratio were still positively associated with impaired renal function, whereas associations were attenuated in GG carriers. The adjusted HRs comparing tertile 3 with tertile 1 for impaired renal function were: AA/AG carriers—zinc 1.359 (1.055–1.749); zinc/copper ratio 1.642 (1.267–2.128); GG carriers—zinc 1.065 (0.623–1.819); zinc/copper ratio 1.584 (0.889–2.822).

**Table 4:** The association between plasma metal and impaired renal function according to gene polymorphism.

Variables	Quartiles of plasma metals’ concentration			*P* for trend	*P* for interaction
	T1	T2	T3		
*Zinc*					
GG	1	0.901 (0.508, 1.596)	1.065 (0.623, 1.819)	0.786	0.006
AA+AG	1	1.046 (0.801, 1.367)	1.359 (1.055,1.749)	0.014	
*Copper*					
GG	1	0.804 (0.478, 1.352)	0.555 (0.307, 1.003)	0.050	0.333
AA+AG	1	0.882 (0.686, 1.133)	0.733 (0.563, 0.954)	0.020	
*Zinc/Copper*					
GG	1	1.484 (0.816, 2,698)	1.584 (0.889, 2.822)	0.138	<0.001
AA+AG	1	1.125 (0.855, 1.479)	1.642 (1.267, 2.128)	<0.001	

### 3.5 Predictive value of zinc/copper ratio, zinc and copper in amputation events for impaired renal function

**Figure 1:**
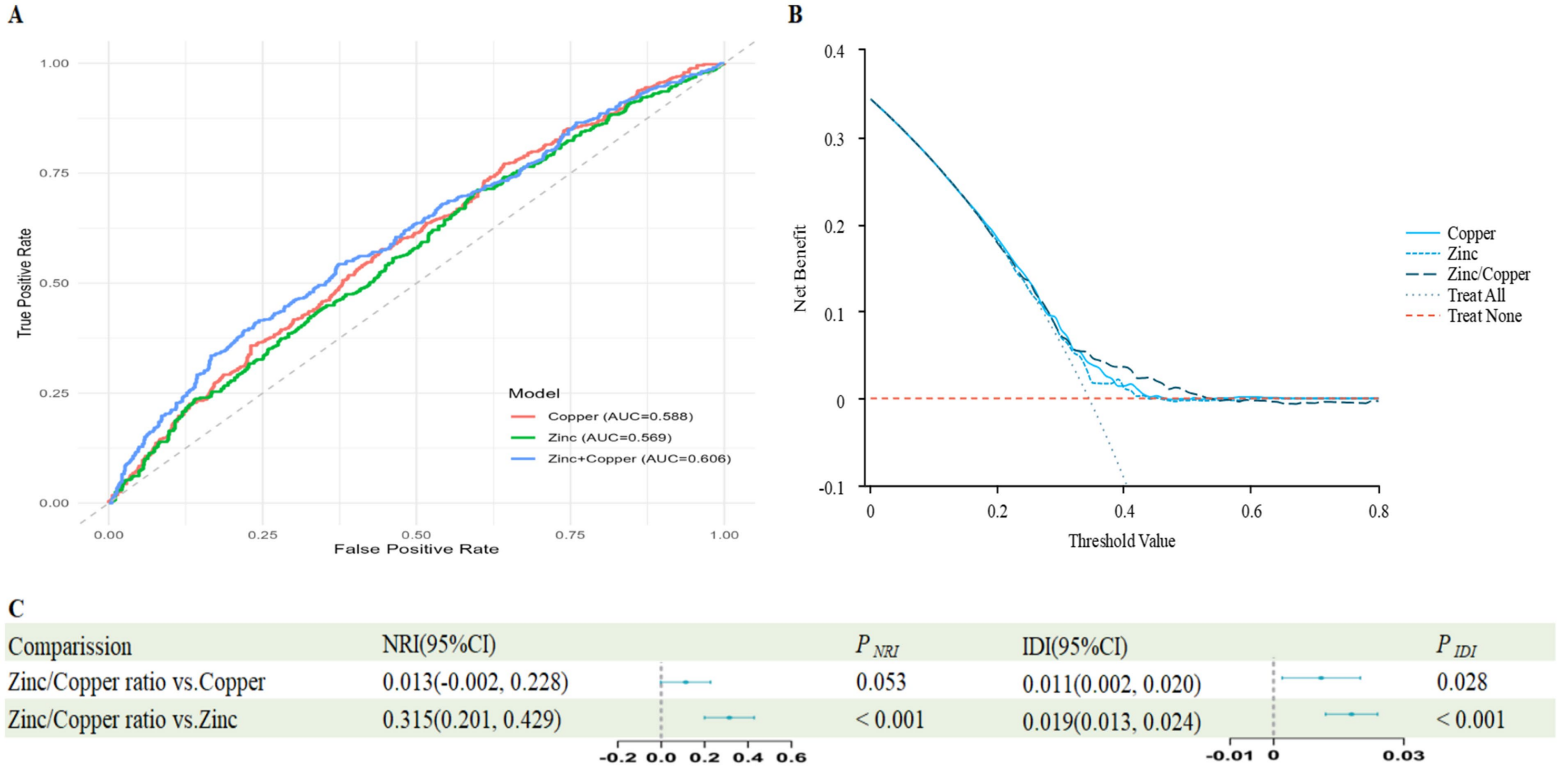
Predictive value of Zinc/Copper ratio, Zinc and Copper for impaired renal function. **(A)** The receiver operating characteristic (ROC) curve evaluating the discriminative capabilities by calculating the AUC; **(B)** Decision curve analysis to compare the clinical utility, the y-axis represents net benefits, calculated by subtracting the relative harm (false positives) from the benefits (true positives). The *x*-axis calculates the threshold probability; **(C)** NRI and IDI index for Zinc/Copper ratio.

The ROC analysis yielded an AUC of 0.606 for the Zinc/Copper ratio (Figure 1A), and the decision curve confirmed its clinical relevance (Figure 1B). Compared with the individual predictors, the Zinc/Copper ratio yielded significant improvement over Zinc on both NRI and IDI, whereas for copper only NRI reached significance (Figure 1C).

## 4 Discussion

To the best of our knowledge, this is the first study to investigate the combined effects of plasma zinc and copper concentrations alongside the superoxide dismutase (SOD1) gene (rs2070424) polymorphism on impaired renal function. Our findings indicate that impaired renal function is positively associated with plasma zinc concentrations and the zinc/copper ratio, while inversely associated with plasma copper levels. Furthermore, the risk allele A of the SOD1 rs2070424 polymorphism was found to be linked to impaired renal function. Notably, significant interactions were observed between the SOD1 rs2070424 variant and plasma zinc, as well as zinc/copper ratio, in relation to impaired renal function.

Prior cross-sectional analysis has reported positive association between higher zinc exposure and impaired renal function in specific settings (23). However, in longitudinal cohorts of patients with established CKD, serum zinc declines as disease progresses, and lower zinc predicts faster progression (11, 24), which differs from the direction observed in our prospective cohort. We interpret this divergence as reflecting disease-related shifts in renal handling: as GFR falls, fractional excretion of filtered solutes increases, generating trace-element imbalance in established CKD (25). Accordingly, assessing zinc prior to renal impairment—as in our cohort—helps clarify the direction of association and underscores the value of our prospective design.

Regarding copper, prior evidence is still inconsistent. Although a case–control study suggested that regulating copper blood concentrations could serve as a potential therapeutic target for preventing and treating diabetic nephropathy (26), whereas several epidemiologic analyses have reported positive associations between plasma copper and impaired renal function (9, 27). Mechanistically, copper is required for SOD1 maturation, lower copper has been linked to reduced SOD1 activity (16, 28, 29), and copper dysregulation can increase reactive oxygen species and oxidative damage (30). This framework is compatible with nonlinear patterns reported for dietary intake (31). Taken together, our data are consistent with a physiologic-range interpretation: adequate copper may support antioxidant defense, whereas dysregulation or excess may be harmful.

Notably, excess intake of either zinc or copper can also disrupt Zn–Cu balance via competitive intestinal absorption and downstream shifts in circulating levels (32, 33). The resulting disequilibrium can compromise antioxidant enzyme activity (34) and foster chronic oxidative stress, increasing the risk of impaired renal function (35). Accordingly, we introduced the zinc/copper ratio as a composite index of metal balance for our analyses. Its use and reliability have been noted in prior studies (13, 36). In our cohort, the zinc/copper ratio—used as a composite indicator—showed a higher AUC than either metal alone for predicting impaired renal function, providing partial evidence of incremental value. Taken together, these considerations highlight the importance of explicitly considering zinc–copper balance when assessing the renal effects of either element in future studies.

In terms of genetic effects, prior studies link the rs2070424 A allele to reduced SOD1 mRNA in immortalized lymphoblastoid cell lines (37) and to lower SOD1 activity relative to GG in Mexican women (38), indicating a weaker antioxidant defense. Consistent with this, in our cohort, the associations of zinc and zinc/copper ratio with impaired renal function were still significant in A-allele carriers and attenuated in GG, with a significant multiplicative interaction. These observations are consistent with a mechanism in which lower SOD1 activity in A-allele carriers heightens susceptibility to oxidative injury, contributing to the observed heterogeneity. Our findings therefore emphasize the need to consider functional genetic effects when evaluating the renal impact of zinc and copper.

This study has both strengths and limitations that should be considered. We assessed the relationship between plasma zinc and copper levels, both individually and as zinc/copper ratio, with the risk of renal impairment. Beyond association, we also evaluated the predictive performance of zinc/copper ratio. Accordingly, our study highlights that investigations of zinc or copper and kidney outcomes should explicitly consider zinc–copper balance, rather than evaluating each element in isolation. Additionally, for the first time, we incorporated gene–environment interactions in our analysis. However, the observational design of the “135” cohort limits our ability to establish causality. Outcome status was ascertained only at two examinations (4 years apart); thus endpoints are interval-censored, and summarizing events over this window may introduce bias. Plasma zinc and copper were measured once at baseline, which may cause exposure misclassification, although lifestyle stability likely mitigates short-term variability. Finally, because the cohort is based in Suzhou, external validity and potential population differences in rs2070424 merit evaluation; external validation is warranted.

## 5 Conclusion

Our results indicated that higher plasma zinc and zinc/copper ratio were positively associated with the risk of impaired renal function, whereas higher copper was inversely associated. These associations were modified by the SOD1 polymorphism. Taken together, considering zinc/copper ratio provides a more informative summary than single-metal measures and shows partial incremental discrimination. Our findings offer new insights into the role of zinc/copper ratio in renal impairment and may help clarify the discrepancies observed in previous research.

## Data Availability

The raw data supporting the conclusions of this article will be made available by the authors, without undue reservation.

## Footnotes

1
http://www.ncbi.nlm.nih.gov/SNP
